# GC-MS-Based Comparative Analysis of Compounds in Host Plants and Insect Gut Extracts

**DOI:** 10.3390/metabo16060351

**Published:** 2026-05-24

**Authors:** Rita Dill, Kimberly Smith, Shelia Okoth, Xavier Cheseto, Anne Osano

**Affiliations:** 1 Department of Natural Sciences, College of Arts and Sciences, Bowie State University, Bowie, MD 20715, USA; rdill@bowiestate.edu (R.D.); smithk0829@students.bowiestate.edu (K.S.); 2 Department of Biology, College or Science and Technology, University of Nairobi, Riverside Drive, Nairobi P.O. Box 30197-00100, Kenya; sheilaokoth@uonbi.ac.ke; 3 International Centre of Insect Physiology and Ecology (*icipe*), Duduville Campus, Thika Road, Kasarani, Nairobi P.O. Box 30772-00100, Kenya

**Keywords:** GC-MS, plant–insect interactions, insect guts, fall armyworm, desert locust, silkworm

## Abstract

**Background/Objectives**: Herbivorous insects feed on plant tissues to obtain nutrients necessary for growth and development while simultaneously ingesting diverse plant secondary metabolites. Understanding the fate of these compounds during digestion is important for advancing knowledge of insect nutritional physiology and diet-associated biochemical processes. This study aimed to comparatively profile metabolites in host plants and corresponding insect gut extracts to generate insights into compound transfer and compositional changes within these systems. **Methods**: Gas Chromatography-Mass Spectrometry (GC-MS) metabolomics was combined with Ultraviolet-Visible (UV–Vis) quantification of total phenols and flavonoids to compare host plant tissues and insect gut extracts in three systems: fall armyworm (*Spodoptera frugiperda*) larvae on maize (*Zea mays*), desert locust (*Schistocerca gregaria*) on wheatgrass (*Triticum aestivum*), and silkworm (*Bombyx mori*) on mulberry (*Morus alba*). The analytical approach targeted semi-volatile and moderate polar compounds within the constraints of the extraction and detection workflow. **Results**: UV–Vis analysis revealed consistent enrichment of total phenols in insect guts relative to host plants (1.4- to 0.35-fold), while flavonoids were reduced (2- to 7-fold). GC-MS analyses showed clear separation of gut and plant metabolomes, with <35% shared metabolites and the majority unique to insect guts. Insect extracts were enriched in hydrocarbons, fatty acids, sterols, and terpenoid derivatives, reflecting extensive biochemical transformation. Sex-specific metabolite differences were observed in silkworm and desert locust guts despite identical diets. These findings show differences between plant and gut metabolite profiles, reflecting selective enrichment, depletion, and restructuring of dietary compounds during digestion. Overall, this study provides comparative metabolic data on insect–plant feeding systems and highlights the gut as a dynamic environment associated with changes in dietary metabolite composition. These findings contribute to understanding how plant-derived compounds are represented in insect gut extracts and establish a baseline framework for future studies investigating the biochemical processes underlying insect digestion and nutrient utilization.

## 1. Introduction

Phytophagous insects encounter complex chemical landscapes when feeding on host plants [1,2]. Plants produce diverse primary and secondary metabolites, including phenols, flavonoids, terpenoids, sterols, fatty acids, and sugars, that function in defense, signaling, and structural integrity [3,4,5]. Among these, phenolic and flavonoid compounds are important due to their roles in anti-herbivore defense, oxidative stress modulation, and deterrence of feeding [6,7]. Successful herbivory therefore requires insects to tolerate, detoxify, sequester, or biochemically transform these compounds [8,9,10]. From a nutritional physiological perspective, ingested plant compounds are processed within the insect gut alongside other dietary constituents such as proteins, lipids, and carbohydrates, which are enzymatically broken down and selectively absorbed during digestion. This general principle of digestive processing is well established across animal systems, where intestinal metabolism governs nutrient availability, compound transformation, and excretion of dietary metabolites. In insects, previous studies have shown that gut physiology not only facilitates nutrient assimilation but also mediates the fate of plant-derived secondary metabolites, although most evidence remains fragmented across targeted biochemical studies rather than integrated in comparative metabolomics approaches [8,11,12,13].

Previous nutritional studies in phytophagous insects have largely focused on digestive enzyme activity, nutrient utilization efficiency, detoxification pathways, gut microbiota, and targeted analyses of specific plant allelochemicals such as phenolics, alkaloids, and terpenoids [13,14]. Other studies have demonstrated that dietary composition can substantially alter insect lipid metabolism, sterol assimilation, antioxidant status, growth performance, and feeding behavior [15,16]. In lepidopteran and orthopteran insects in particular, host plant chemistry has been associated with differences in nutrient conversion efficiency, digestive physiology, and metabolic adaptation to chemically diverse diets [17,18,19]. However, despite these advances, relatively few studies have systematically compared whole metabolite profiles between host plant tissues and corresponding insect gut extracts under standardized feeding conditions using untargeted analytical approaches. Therefore, the extent to which gut metabolite composition reflects direct dietary intake versus digestion-associated biochemical restructuring remains insufficiently characterized.

While the biochemical mechanisms of insect detoxification, including cytochrome P450 monooxygenases, glutathione-S-transferases, and esterases, are well documented, and the role of gut-associated microbiota in insect nutrition and metabolism is increasingly recognized [8,9,10], direct comparative analyses of host plant tissues and insect gut metabolomes under controlled feeding conditions remain limited. Existing studies show that gut environments influence the breakdown, modification, and absorption of dietary compounds, including plant secondary metabolites, but these processes are often examined in isolation or within single-species systems rather than across multiple feeding strategies.

Recent advances in metabolomics have enabled holistic characterization of diet-associated metabolic signatures in biological systems; however, applications in insect nutritional physiology remain comparatively limited, particularly in studies integrating both host plant and insect gut metabolomes [20]. Comparative metabolomic profiling therefore offers complementary insight beyond conventional nutritional assays by identifying metabolite enrichment, depletion, and compositional divergence associated with digestion and dietary utilization. A systematic, untargeted comparison is therefore necessary to determine whether gut metabolomes primarily reflect ingested plant chemistry or represent digestion-associated alterations in chemical composition. Such comparisons are relevant for agriculturally important pests, where dietary flexibility and utilization of different host plants may contribute to feeding performance and adaptation. Gas Chromatography–Mass Spectrometry (GC-MS) analysis can provide a platform for characterizing volatile and semi-volatile metabolites relevant to plant–insect interactions, including fatty acids, hydrocarbons, sterols, and terpenoid-derived compounds [21]. When combined with quantitative spectrophotometric assessment of total phenols and flavonoids, this approach enables profiling and targeted evaluation of key defensive metabolite classes. Together, these complementary techniques allow direct assessment of compositional overlaps, fold-change differences, and the proportion of shared versus unique metabolites between plants and insect guts [22,23].

These systems were selected because they represent biologically and agronomically distinct feeding relationships involving economically important insect and crop species. The fall armyworm is a highly destructive polyphagous pest of major cereal crops, the desert locust is a broad generalist herbivore with substantial agricultural impact, while the silkworm is a specialist feeder with a long history of domestication and dependence on mulberry foliage. Collectively, these species, all obtained from protocols, allow assessment of the differences in metabolite profiles associated with insect feeding ecology; therefore, the coverage represents a partial chemical profile of the systems analyzed. Here, we conducted a comparative metabolomic analysis across three agriculturally relevant plant–insect systems: fall armyworm (*Spodoptera frugiperda*) feeding on maize (*Zea mays*), desert locust (*Schistocerca gregaria*) feeding on wheatgrass (*Triticum aestivum*), and silkworm (*Bombyx mori*) feeding on mulberry (*Morus alba*). Three systems were established rearing at the international centre of insect physiology and ecology, ensuring controlled feeding conditions and consistent sample quality, as well as representing comparative gradient feeding strategies, such as polyphagous, generalist, and specialist. By profiling host plant tissues prior to feeding and dissecting gut extracts after controlled feeding, we quantified metabolic divergence using GC-MS and assessed fold differences in total phenolic and flavonoid using Ultraviolet-Visible (UV–Vis) spectrophotometry. We evaluated whether insect sex contributes to gut metabolomic in species where males and females were analyzed separately. While our work follows practices of untargeted metabolomics, it is necessary to highlight that the compounds analyzed are restricted by the nature of the GC-MS instrumentation and the solvents used.

## 2. Materials and Methods

### 2.1. Chemicals

Cholesterol (≥99%) was obtained from BDH Chemicals Ltd, Poole, Dorset, (UK), while stigmasterol (~95%) and campesterol (~65%, crystalline) were purchased from Sigma-Aldrich (St. Louis, MO, USA). All the other chemicals/certified reference materials were sourced from Merck, Darmstadt, Hesse, (Germany). This included dichloromethane (GC-grade); hydrocarbons (C_5_–C_30_); straight-chain alkanes; α-pinene; methyl 9*Z*,12*Z*,15*Z*-octadecatrienoate; phytol; 9*Z*,12*Z*,15*Z*-octadecatrienoic acid; octadecanoic acid; C20-ol; squalene; vitamin E; and α-cedrene, with purities ranging from 90% to 99.9%.

### 2.2. Plants

Three plant species, maize (*Zea mays* L.), wheatgrass (*Triticum aestivum* L.), and mulberry (*Morus alba* L.), were selected based on their use as host plants for the rearing of desert locust (*Schistocerca gregaria* Forsskål, 1775, Orthoptera: Acrididae), fall armyworm (*Spodoptera frugiperda* J.E. Smith, 1797, Lepidoptera: Noctuidae), and silkworm (*Bombyx mori*, Linnaeus, 1758, Lepidoptera: Bombycidae), respectively, at the International Centre of Insect Physiology and Ecology (*icipe*), Nairobi, Kenya. Maize and wheat were cultivated in May 2025.

Maize hybrid “SC Duma 43” (Seed Co., Nairobi, Kenya) was cultivated in an 80 × 30 ft field plot at *icipe*, Duduville campus (1.2219° S, 36.8967° E; 1616 m above sea level). Seeds were sown at a rate of two seeds per hole, spaced 35 cm apart, and watered daily under natural environmental conditions. The plants were harvested at four weeks of age for use in experiments.

Wheat seeds, Robin variety, were procured from a local market in Nairobi and grown in a screenhouse at *icipe* in 2 L plastic pots, as described [21]. The plants were maintained under natural light conditions and watered daily. At four weeks of age, whole plants were uprooted and used for the experiments.

Mature mulberry leaves (white mulberry, *Morus alba*, Ichinose, variety) were harvested from the *icipe* botanical garden, where the plants are continuously cultivated for silkworm rearing. The mulberry plants are grown under rain-fed conditions without supplemental irrigation. No synthetic fertilizers were applied; instead, standard agronomic practices were followed, including manual weeding and the monthly application of insect frass fertilizer sourced from our black soldier fly rearing facility. The frass was used as an organic nutrient amendment to support leaf production for silkworm feeding that are reared within *icipe* campus. Only healthy, fully expanded leaves were collected for use in the experiments.

### 2.3. Plant Collection and Preparation

Fresh plant materials of maize (*Zea mays*; hybrid “SC Duma 43”), wheatgrass (*Triticum aestivum*), and mulberry (*Morus alba*) were collected in the morning (10–11 am) from *icipe* farm screenhouse and field plots as appropriate. In all cases, only visibly healthy and undamaged leaves were selected to minimize variation associated with induced plant defense volatiles. The samples were weighed, oven-dried at 55 °C for 72 h, and ground to a fine powder using a mortar and pestle. While liquid nitrogen-based cryogenic processing could better preserve highly labile and volatile metabolites, oven-drying was used to ensure sample stability, uniform moisture removal, and compatibility with downstream GC-MS analysis of semi-volatile compounds. This introduces a potential limitation in capturing the full metabolite spectrum, which is acknowledged in the study.

### 2.4. Extraction

For each plant, 10 g of powdered material was placed into 15 mL Falcon tubes and extracted with 13 mL of methanol (LC-MS grade, Sigma-Aldrich, St. Louis, MO, USA). The mixtures were vortexed for 10 s, sonicated for 15 min, and cold-macerated in the dark for 72 h with daily agitation. Extracts were filtered using Whatman (GE Whatman, Maidstone, Kent, UK) No. 1 filter paper, and methanol was evaporated under reduced pressure. Extraction yield was determined gravimetrically. For analysis, 50 mg of each dried extract was reconstituted in 1 mL of 50% methanol, vortexed, sonicated, and filtered.

### 2.5. Insects

#### 2.5.1. Fall Armyworm (*Spodoptera frugiperda*)

Fifth instar fall armyworm larvae were obtained from the ARQU at *icipe.* The colony was originally established using fall armyworm collected from maize fields in Mbeere, Embu County, Kenya (00°42′25.1″ S, 037°29′0.14″ E; 1091 m a.s.l.). Larvae were reared on fresh maize leaves, sourced from *icipe*’s on-station farm, inside well-ventilated, sleeved Perspex cages (60  ×  60  ×  60 cm), following the rearing protocol described in the literature [24]. Leaves were replaced every three days to ensure a consistent food supply. Paper towels lined the cage floor to absorb moisture and create favorable conditions for pupation. Once pupation occurred, pupae were transferred to cylindrical plastic containers (10 mm diameter × 50 mm height, Kenpoly, Nairobi, Kenya) lined with cotton wool and housed in ventilated Perspex cages (30  ×  30  ×  30 cm) until adult emergence to sustain colony development. For the metabolomic analyses, fifth instar larvae were used; however, due to the difficulty in reliably distinguishing males from females at this developmental stage, samples were treated as a single pooled group without sex differentiation.

#### 2.5.2. Desert Locust (*Schistocerca gregaria*)

Gregarious-phase desert locusts were reared at *icipe* within the Insect and Animal Rearing and Quarantine Unit (ARQU). The insects were maintained on a diet of wheatgrass seedlings and wheatgrass bran in a controlled environment chamber set at 30 ± 4 °C, 40–50% relative humidity (RH), and a 12:12 h light to dark photoperiod. Approximately 200–250 insects were housed in aluminum cages (50 × 50 × 50 cm) within a dedicated, well-ventilated room (4.5 × 4.5 m) equipped with a duct system to maintain negative pressure. Fifth instar nymphs were collected and immediately euthanized on ice for further processing.

#### 2.5.3. Silkworm (*Bombyx mori*)

Silkworms were reared on mulberry leaves at *icipe* according to standardized rearing protocols. Fifth instar larvae were collected and prepared for experimentation.

### 2.6. Insect Dissection and Gut Sample Preparation

Following collection, all insects were euthanized by cold immobilization on ice. The gut tissues, along with their contents, were aseptically dissected from each insect. Individual gut samples were transferred into 1 mL of sterile saline solution, then homogenized using a glass rod. The homogenates were vortexed for 10 s, sonicated for 15 min, and centrifuged at 4700 rpm for 10 min. The resulting supernatants were mixed with an equal volume of 100% methanol and stored at 4 °C prior to quantitative analysis of total flavonoid content and total phenolic content.

### 2.7. Quantitative Phytochemical Analysis

Quantitative analysis of total flavonoid content (TFC) and total phenolic content (TPC) was carried out for both plant tissues and insect gut extracts according to the published protocols [25,26].

### 2.8. Total Flavonoid Content (TFC)

TFC was determined using an aluminum chloride colorimetric method. Briefly, 1 mL of 50% methanol extract (50 mg/mL) was mixed with 4 mL of 50% methanol, followed by 0.3 mL of 5% sodium nitrite (NaNO_2_). After 5 min, 0.3 mL aluminum chloride (10%, AlCl_3_) was added. After 1 min, 2 mL of 1 M sodium hydroxide (NaOH) and 2.4 mL of 50% methanol were added. Absorbance was measured at 510 nm using a Jenway 6850 UV/Vis spectrophotometer (manufactured by Cole-Parmer Ltd. (Jenway), Stone, Staffordshire, UK). A blank (without extract) was used as control. A standard calibration curve was prepared using quercetin (20–500 µg/mL), and results were expressed as micrograms of quercetin equivalents per milliliter (µg QE/mL).

### 2.9. Total Phenolic Content (TPC)

TPC was determined using a modified Folin–Ciocalteu method. A 1 mL aliquot of 50% methanol extract (50 mg/mL) was mixed with 5 mL of 0.2 N Folin–Ciocalteu reagent and incubated for 5 min. Then, 4 mL of sodium carbonate solution (75 g/L) was added, and the mixture was left to react at room temperature for 1 h. Absorbance was measured at 760 nm using a Jenway 6850 UV/Vis spectrophotometer. A blank control (water in place of extract) was included. A gallic acid standard curve (0–500 µg/mL) was used to calculate phenolic content, and was expressed as micrograms of gallic acid equivalents per milliliter (µg GAE/mL).

### 2.10. GC-MS: Gas Chromatography–Mass Spectrometry

The extracts were allowed to evaporate until dry under a fume hood and subsequently reconstituted (100 mg) in 500 µL of GC-grade dichloromethane (DCM) (Sigma-Aldrich, St. Louis, MO, USA). The solutions were vortexed for 10 s, sonicated for 10 min, and centrifuged at 14,000 rpm for 5 min. The resulting supernatant was dried over anhydrous Na_2_SO_4_, and an aliquot (1.0 µL) was analyzed by GC-MS using a 7890A gas chromatograph (Agilent Technologies, Inc., Santa Clara, CA, USA) coupled to a 5975C mass selective detector (Agilent Technologies, Inc., Santa Clara, CA, USA). The GC system was equipped with a low-bleed (5%-phenyl)-methylpolysiloxane HP-5MS capillary column (30 m × 0.25 mm i.d., 0.25 µm film thickness; J&W, Folsom, CA, USA). Helium was used as the carrier gas at a constant flow rate of 1.25 mL/min. The injector temperature was maintained at 270 °C, while the transfer line temperature was set at 280 °C. The oven temperature program was as follows: initial temperature of 35 °C held for 5 min, increased at 10 °C/min to 280 °C, and held for 20.4 min [27].

The mass selective detector was operated under electron impact (EI) ionization at 70 eV, with the quadrupole and ion source temperatures maintained at 180 °C and 230 °C, respectively. Mass spectra were acquired in full-scan mode over an m/z range of 40–550, with a solvent delay of 3.3 min. Blank runs of the reconstituting solvents (DCM and evaporated MeOH) and instrument blanks were analyzed under identical conditions, and their corresponding peaks were excluded from the analysis. Compound identification was based on a comparison of retention times and mass fragmentation patterns with those of authentic reference standards where available, as well as reference spectra from the National Institute of Standards and Technology (NIST) mass spectral libraries (versions 05, 08, and 11). The following compounds were definitively identified via authentic standards; cholesterol; stigmasterol; campesterol; hydrocarbons (C_5_–C_30_); straight-chain alkanes; α-pinene; methyl 9*Z*,12*Z*,15*Z*-octadecatrienoate; phytol; 9*Z*,12*Z*,15*Z*-octadecatrienoic acid; octadecanoic acid; C20-ol; squalene; vitamin E; and α-cedrene.

### 2.11. Data Analysis

Non-metric multidimensional scaling (NMDS) was used to display differences in the metabolite composition between our plant extracts and the insect gut samples. Dissimilarity matrices were generated using Bray–Curtis distance, and NMDS ordinations were executed in two dimensions. Stress values for each ordination were calculated to assess goodness of fit. Lower stress values signaled better preservation of rank order distances in a reduced, dimensional space. Pairwise NMDS analyses were conducted for each plant and its corresponding insect. Replicates were plotted, and overlaid with the grouped centroids with ±1 standard deviation to show dispersion.

The Jaccard similarity indices were determined for each plant and insect system by dividing the number of unique distinct metabolites detected amongst the two combined sets of data. This was done using presence–absence matrices that were restricted to compounds observed in all replicates. “Presence” was defined as the detection of a compound in at least two replicates within a group. The denominator’s total metabolite count did not include any duplicates in detections and came from the plant and insect system metabolite lists. When metabolites were present in both groups, data was normalized for the relative GC-MS peak areas to the total ion signal within each sample. Subsequently, averages were taken across the replicates. Fold differences were calculated via comparing the mean normalized abundance from the insect gut extracts with the corresponding plant. The metabolites were considered depleted when the mean relative abundance decreased in the insect gut. The metabolites were considered enriched when the mean relative abundance increased in the insect gut. All analyses and visualizations were performed using Excel or Python-based (version 3.11.15) workflows incorporating Pandas (version 3.0), SciPy (version 1.17.0), NumPy (version 2.4), and Matplotlib (version 3.10).

## 3. Results

### 3.1. Insect Gut and Host Plant Extract Yield

**Figure 1:**
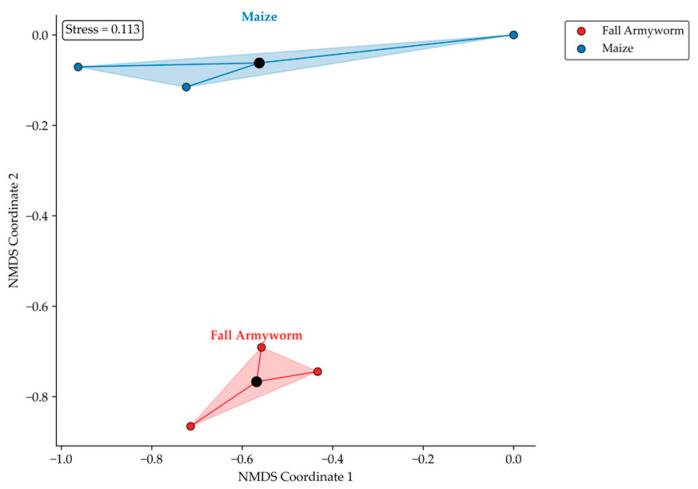
Non-metric multidimensional scaling ordination of GC-MS-detected metabolite profiles from maize leaves and fall armyworm gut extracts. The ordination was generated using Bray–Curtis dissimilarity calculated from detected compound profiles. Each point represents an individual replicate, and colors indicate sample type. The blue color represents the maize, and the red color represents the fall armyworm. Group centroids are shown with ±1 standard deviation to illustrate within-group dispersion. The separation between maize and fall armyworm gut samples indicates distinct metabolite composition between the host plant and corresponding insect gut extract. NMDS stress = 0.113.

**Figure 2:**
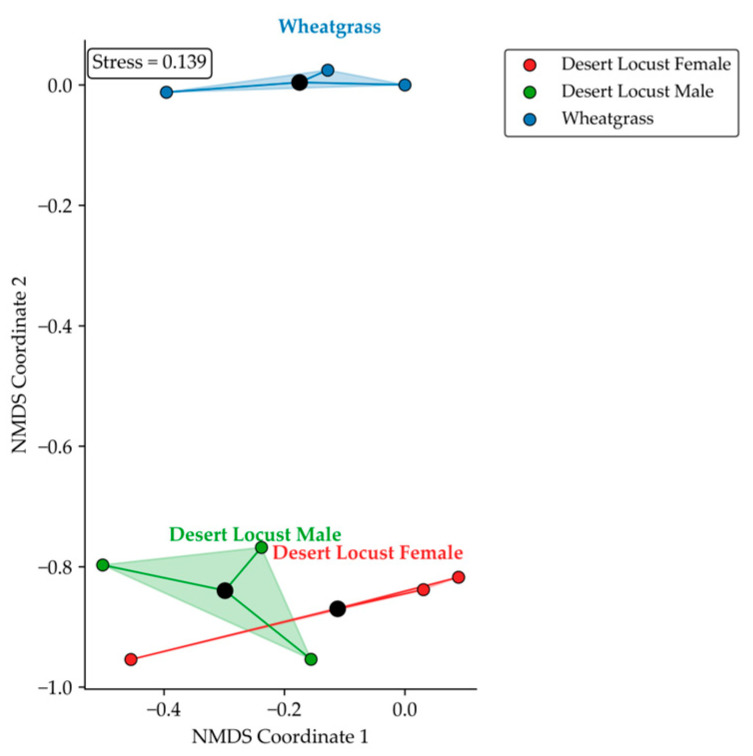
Non-metric multidimensional scaling ordination of GC-MS-detected metabolite profiles from wheatgrass and desert locust gut extracts. Bray–Curtis dissimilarity was used to compare metabolite composition among wheatgrass, male desert locust gut, and female desert locust gut samples. Each point represents an individual replicate, with varying colors indicating different sample groups. Blue color represents the wheatgrass, red represents the female group of the desert locust, and green represents the male group of the desert locust. Group centroids with ±1 standard deviation shows the dispersion of replicates within each group. The ordination shows compositional separation between wheatgrass and desert locust gut extracts, with male and female gut samples plotted separately. NMDS stress = 0.139.

**Figure 3:**
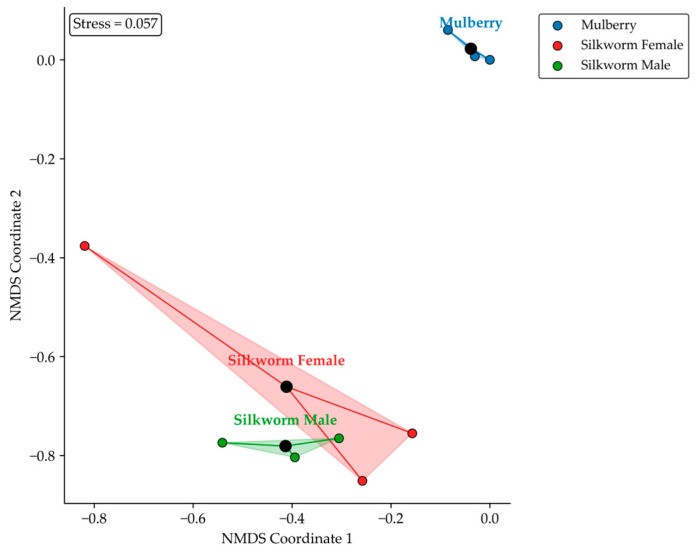
Non-metric multidimensional scaling ordination of GC-MS-detected metabolite profiles from mulberry leaves and silkworm gut extracts. Bray–Curtis dissimilarity was used to compare metabolite composition among mulberry, male silkworm gut, and female silkworm gut samples. Points represent individual replicates, and colors indicate sample groups. The blue color corresponds to the mulberry plant. The red color represents the female group of the silkworm, and the green color represents the male group of the silkworm. Group centroids with ±1 standard deviation indicate within-group dispersion. The ordination shows separation between mulberry leaf extracts and silkworm gut extracts, indicating distinct metabolite profiles between the host plant and insect gut. NMDS stress = 0.057.

The extraction yields from insect guts and corresponding host plants have been reported throughout the manuscript in Figure 1, Figure 2 and Figure 3 and the Supplementary Excel Files. In the fall armyworm feeding on maize, the gut mass accounted for less than half of the total body mass and produced a gut extract yield of 0.01 g, while the maize plant extract yield was 0.98 g. In the silkworm reared on mulberry, the gut constituted approximately 40–60% of total body mass irrespective of sex, with a gut extract yield of 0.2 g compared to a mulberry extract yield of 1.25 g. Similarly, in the desert locust fed on wheatgrass, the gut represented less than one-third of total body mass in both sexes and yielded 0.2 g of extract, whereas wheatgrass yielded 1.09 g.

### 3.2. Metabolite Profiles of Maize Leaves and Fall Armyworm Gut Extracts Analyzed by GC-MS

GC-MS analysis identified a total of 70 metabolites across maize leaves and fall armyworm gut extracts; full compound composition tables and raw GC-MS peak areas are provided in the Supplementary Excel Files. Of these, 15 compounds were detected in maize and 37 in fall armyworm gut extracts, with 18 metabolites shared between matrices. Fifteen compounds were unique to maize, whereas 37 were exclusive to the fall armyworm gut, indicating greater metabolite richness in fall armyworm samples.

Presence–absence analysis revealed limited compositional overlap between matrices, with a Jaccard similarity index of 0.26, indicating that only 26% of detected compounds were shared. The NMDS ordination (Figure 1) showed low-to-moderate similarity, with clear separation between maize and fall armyworm gut samples, confirming distinct metabolomic profiles.

Quantitative comparison of shared metabolites revealed pronounced shifts in relative abundance between maize tissues and fall armyworm gut extracts. Overall, fatty acids, sterols, tocopherols, and triterpenoid-related compounds were relatively enriched in fall armyworm gut extracts, whereas long-chain plant-associated hydrocarbons were reduced. Epicuticular hydrocarbons recorded strong reduction in the fall armyworm gut compared to maize. In contrast, lipid-derived and sterol-related metabolites showed increased relative abundance in the fall armyworm gut extracts. Fold-change values for shared metabolites are presented in Table 1.

### 3.3. Metabolite Profiles of Wheatgrass and Desert Locust Gut Extracts Analyzed by GC-MS

Upon conducting our GC-MS analysis, we were able to identify a total of 64 metabolites across wheatgrass and the desert locust male gut, and 67 metabolites in females (Supplementary Files). In males, 31 compounds were recorded in wheatgrass and 41 in the gut, with eight metabolites shared between the two matrices. Our analysis revealed limited compositional overlap between desert locust gut extract and wheatgrass, as reflected by a Jaccard similarity indices of 0.13 for males and 0.15 for females. The NMDS ordination we generated (Figure 2) showed low-to-moderate similarity. We saw separation notable between wheatgrass and desert locust gut samples, confirming distinct metabolomic profiles.

We performed a quantitative evaluation of the shared metabolites. This evaluation showed the abundance shifts between the plant and gut systems. From this, we noted that sterols, including campesterol, stigmasterol, and β-sitosterol, were depleted in both male and female gut extracts relative to wheatgrass. In contrast, several hydrocarbons (e.g., pentacosane, octacosane, nonacosane) and selected fatty acid derivatives showed relative enrichment in gut samples, especially in females (Table 2).

### 3.4. Metabolite Profiles of Mulberry Leaves and Silkworm Gut Extracts Analyzed by GC-MS

Our GC-MS analysis identified a total of 62 shared compounds between mulberry leaves and the male silkworm gut extract, and 63 shared metabolites in the females. We saw that, in the case of males, 33 compounds came from mulberry plants, 44 from the silkworm gut extracts, and 12 were shared. Similarly, in the female comparison, 21 compounds were unique to the mulberry, while 32 metabolites were exclusive to the gut extract (Supplementary Files). Our analysis revealed limited compositional overlap between mulberry and silkworm gut extract, as reflected by a Jaccard similarity index of 0.18 for both sexes. Our NMDS ordination showed low-to-moderate similarity between the sexes of the silkworm and mulberry samples (Figure 3).

We performed a quantitative comparison of shared metabolites, which revealed differences in relative abundance between the plant and the insect gut. We found that long-chain hydrocarbons, phytol and its derivatives, sterols, and methyl esters were generally depleted in the silkworm gut compared to mulberry leaves. In contrast, some fatty acids, e.g., 9Z,12Z,15Z-octadecatrienoic acid, were enriched in female gut extracts (Table 3).

### 3.5. Flavonoids and Phenolic Compounds in Maize Leaves and Fall Armyworm Gut Extracts Analyzed by UV–Vis Spectroscopy

**Figure 4:**
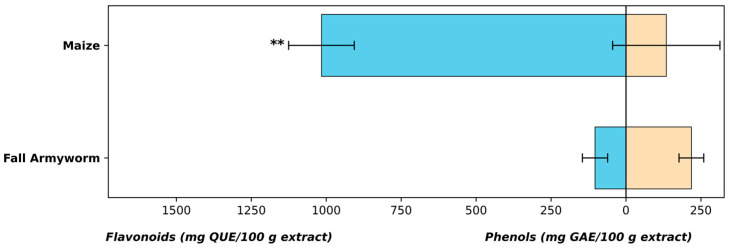
Total phenol and flavonoid concentrations present in the fall armyworm gut and maize plant extracts. Blue-colored bars correspond to the flavonoid samples in units of mg QUE per 100 g extract. Yellow-colored bars represent the phenol samples in units of mg GAE per 100 g extract. Error bars are used to depict standard deviation across the three replicates. Asterisks denote statistically significant differences; ** *p* < 0.01, as determined by one-way ANOVA. Here, we report our asterisks correspond to a flavonoid *p*-value of 0.0018.

**Figure 5:**
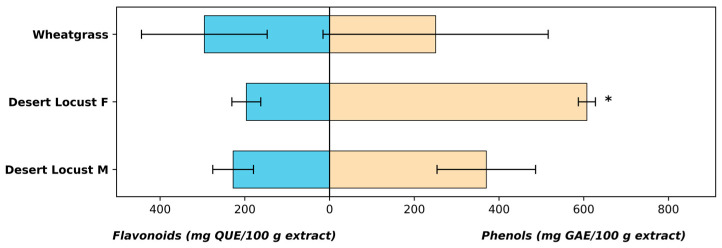
Bar chart shows the phenol concentrations present in the desert locust gut and wheatgrass plant extracts. F = females, M = males. Blue-colored bars correspond to the flavonoid samples in units of mg QUE per 100 g extract. Yellow-colored bars represent the phenol samples in units of mg GAE per 100 g extract. Error bars are used to depict standard deviation across the three replicates. Asterisks denote statistically significant differences; * *p* < 0.05, as determined by one-way ANOVA. Here, we report our asterisks correspond to a phenol *p*-value of 0.0214.

**Figure 6:**
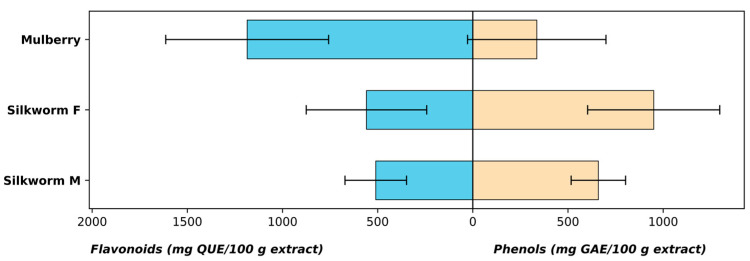
Phenol concentrations present in the silkworm gut and mulberry plant extracts. F = females, M = males. Blue-colored bars correspond to the flavonoid samples in units of mg QUE per 100 g extract. Yellow-colored bars represent the phenol samples in units of mg GAE per 100 g extract. Error bars depict standard deviation across the three replicates. We report no statistically significant differences.

From our Ultraviolet–Visible spectroscopy (UV–Vis) data, we observed that there were lower flavonoid levels (Figure 4) in the fall armyworm (77.66 mg QUE/100 g) when compared to maize leaves (507.89 mg QUE/100 g). This corresponded to a fold change of 0.15 (Log_2_FC = −2.71). Phenolic compounds were higher in fall armyworm (1027.24 mg GAE/100 g) than in maize leaves (598.38 mg GAE/100 g) and possessed a fold change of 1.72 (Log_2_FC = 0.78). A similar trend was observed for wheatgrass–desert locust system (Figure 5) and mulberry–silkworm system (Figure 6).

## 4. Discussion

Phytophagous insects interact with chemically complex host plants containing diverse primary and secondary metabolites that influence growth, defense, and ecological interactions [1,2,3]. In this study, we compared GC-MS-detectable semi-volatile compounds and UV–Vis-quantified phenolic classes between host plant tissues and insect gut extracts across three representative systems: maize–*Spodoptera frugiperda*, wheatgrass–*Schistocerca gregaria*, and mulberry–*Bombyx mori*. The objective was not to reconstruct a complete metabolome, but to generate a comparative chemical profile of ingested plant compounds and their transformation within insect digestive systems under controlled feeding conditions.

The recorded body masses of *Spodoptera frugiperda*, *Schistocerca gregaria*, and *Bombyx mori*, were within established physiological ranges, confirming that all specimens were developmentally normal and suitable comparative metabolomic analysis [2,4,28]. The proportional gut mass relative to total body weight further indicates active feeding prior to dissection, which ensured that the gut metabolite profiles reflected recently ingested host material rather than starvation-induced metabolic shifts [29]. However, gut extracts also represent a composite of ingested plant metabolites, insect-derived enzymatic transformation products, and gut microbiota contributions, meaning that observed differences reflect integrated biochemical outcomes rather than direct transfer of plant compounds. Additionally, this study does not measure detoxification at enzymatic or gene expression levels. Detoxification is therefore inferred only from compositional shifts (e.g., flavonoid reduction and emergence of modified phenolics and lipid derivatives) rather than directly demonstrated. Similarly, “metabolic adaptation” refers strictly to observed chemical profile differences and not physiological or molecular adaptation.

Methanolic extraction produced reproducible metabolites from both plant tissues and insect gut matrices for GC-MS-based profiling. As expected, plant tissues yielded higher crude extract quantities due to greater biomass and structural complexity; however, reliable detection of metabolites in low-biomass gut samples confirms adequate analytical sensitivity. While this study provides insight into metabolites detectable by GC-MS, it is important that future work incorporates complementing analytical platforms, such as UHPLC-MS/MS, LC-Qtof-MS, LC-Orbitrap-MS, and NMR, to achieve broader metabolite coverage. Also, the use of solvents of a wider polarity range would likely enhance the detection of less abundant and highly polar compounds. In addition, the differences in extraction efficiency and normalization basis may influence metabolite interpretation, particularly as wet weight normalization of insect gut samples may introduce variability due to moisture content. Together, addressing these limitations would improve analytical resolution and enable a more comprehensive characterization of host–insect metabolic exchange. Future studies should also incorporate recovery tests, use of internal and external standards, labeled biosynthetic approaches, diet manipulation experiments, larger datasets to facilitate different statistical analyses, more replication to better resolve metabolite pathways and improvements to quantitative robustness.

Integrated GC-MS and phytochemical analyses across the maize–*S. frugiperda*, wheatgrass–*S. gregaria*, and mulberry–*B. mori* systems reveal a consistent overarching pattern: insect gut profiles were structurally and quantitatively distinct from their respective host plants. This divergence supports the concept that the insect midgut functions not as a passive conduit but as a dynamic biochemical reactor shaped by enzymatic digestion, selective absorption, detoxification, sterol conversion, and microbial transformation [26,30]. While each host–insect pairing exhibited species-specific nuances, several conserved metabolic signatures emerged across all systems. These patterns suggest active biochemical transformation of ingested plant compounds within insect digestive systems, although no enzymatic assays were performed to directly confirm these processes.

Maize tissues were comparatively rich in flavonoids, whereas fall armyworm gut extracts displayed reduced flavonoid levels and increased total phenolics, consistent with patterns observed in the other systems (Figure 4). *S. frugiperda*, a highly polyphagous lepidopteran, possesses extensive detoxification machinery, including cytochrome P450 monooxygenases, glutathione-S-transferases, and carboxylesterases [31]. The observed depletion of flavonoids likely reflects oxidative degradation and conjugation processes mediated by these enzymatic systems. It is important to note that this comparison integrates dry weight plant extracts and wet weight insect gut samples and should therefore be interpreted as reflecting relative biochemical transformation rather than absolute quantitative equivalence. Future studies should incorporate freeze-drying of samples prior to extraction and TPC and TFC determination, or alternatively rapid snap-freezing using liquid nitrogen, to minimize enzymatic activity and prevent potential metabolite degradation.

GC-MS profiling revealed enrichment of fatty acids such as n-hexadecanoic acid and linolenic acid in gut extracts, underscoring their importance for lipid metabolism and energy production [3,32,33]. Phytosterol depletion, accompanied by cholesterol accumulation, indicates conserved sterol bioconversion, a common feature in insect physiology [24,34,35]. Additionally, selective assimilation of linolenic acid, a precursor of plant jasmonate signaling, may influence plant defense responses during herbivory [36]. Future studies should employ labeled compounds to actively track the metabolic interchange between the plants and insects.

The wheatgrass–desert locust system shows comparable patterns of metabolic restructuring, though quantitatively distinct. Wheatgrass contained moderate levels of phenolics and flavonoids, whereas desert locust gut extracts exhibited elevated total phenolics and reduced flavonoids, particularly in males. This mirrors the silkworm system and supports active flavonoid degradation and oxidative transformation during digestion. Phenolic accumulation in the gut likely represents enzymatic intermediates generated during metabolic processing [9]. However, these interpretations remain speculative due to the absence of hormonal, transcriptomic, or enzymatic validation.

GC-MS profiling revealed depletion of plant sterols within *S. gregaria* gut extracts, accompanied by cholesterol and sterol epoxide formation. These conversions align with known orthopteran sterol metabolism via dealkylation and hydrogenation pathways [34]. Enrichment of hydrocarbons, fatty acids, and long-chain alcohols indicates active lipid mobilization and energetic channeling within the gut. Male desert locusts showed higher total phenolic content than females, suggesting sex-specific metabolic differentiation, potentially linked to hormonal regulation, feeding intensity, or reproductive allocation [10,25]. Female insects often require greater amounts of certain metabolites for their sex-specific biological processes [25]. Contrastingly, males may use specific metabolites for spermatogenesis or mating behaviors [16]. In many insects, hydrocarbons, fatty acids, and terpenoid-derived metabolites are used in pheromone biosynthesis, and this may result in the differences noted [37]. These findings indicate that insect gut metabolomes may also be impacted by sex-specific needs.

Mulberry leaves exhibited high flavonoid content relative to total phenols, reflecting their defensive phytochemical richness. In contrast, silkworm gut extracts, both male and female, showed elevated total phenolic content and reduced flavonoid levels (Figure 6). This pronounced inversion strongly indicates extensive flavonoid transformation within the silkworm midgut. As a specialist feeder co-evolved with mulberry, *B. mori* possesses highly efficient detoxification and digestive systems capable of de-glycosylation, oxidation, and structural modification of flavonoids [38]. The reduction in intact flavonoids alongside increased total phenols likely reflects conversion into simpler phenolic intermediates or oxidative by-products generated during digestion [39,40].

GC-MS data reinforces this interpretation. Mulberry tissues were enriched in phytol, tocopherols, squalene, β-sitosterol, and triterpenes such as α-amyrin, whereas silkworm guts displayed enrichment of fatty acids, including 9Z,12Z,15Z-octadecatrienoic acid, cholesterol, γ-sitosterol, and sterol derivatives. The enrichment of essential fatty acids suggests preferential assimilation for membrane biosynthesis and energy production [3,33]. Concurrently, the prominence of cholesterol and modified sterols reflects the well-documented sterol auxotrophy of insects, which convert dietary phytosterols into cholesterol for membrane stability and ecdysteroid synthesis [5,6,35]. The comparatively higher representation of sterol and triterpenoid derivatives in females may indicate metabolic allocation toward reproductive physiology, especially oogenesis [25].

## 5. Conclusions

This study provides comparative chemical evidence that insect gut systems significantly restructure host plant metabolite profiles, based on GC-MS-detectable semi-volatile compounds and UV–Vis phytochemical quantification. While the results support the insect gut as a dynamic biochemical transformation environment, interpretations remain restricted to relative chemical profiling and do not include direct enzymatic, genetic, or microbiome measurements. These findings establish a baseline framework for future integrative multi-omics studies of plant–insect metabolic interactions.

## Figures and Tables

**Table 1:** Fold-change comparison of shared metabolites in maize and fall armyworm gut extracts.

Compound Name	Insect Gut Sample	Fold Change	Status in Insect Gut
Heptadecane	Fall armyworm gut	0.23	Depleted
Methyl hexadecanoate	Fall armyworm gut	1.07	Enriched
Hexadecanoic acid	Fall armyworm gut	2.31	Enriched
Methyl (9Z,12Z)-octadeca-9,12-dienoate	Fall armyworm gut	0.89	Depleted
Methyl (9Z,12Z,15Z)-octadeca-9,12,15-trienoate	Fall armyworm gut	0.30	Depleted
(2E,7R,11R)-3,7,11,15-tetramethylhexadec-2-en-1-ol	Fall armyworm gut	1.04	Enriched
(9Z,12Z,15Z)-octadeca-9,12,15-trienoic acid	Fall armyworm gut	1.39	Enriched
Octadecanoic acid	Fall armyworm gut	1.42	Enriched
Docosane	Fall armyworm gut	4.60	Enriched
Tricosane	Fall armyworm gut	0.01	Depleted
Tetracosane	Fall armyworm gut	0.31	Depleted
Pentacosane	Fall armyworm gut	0.26	Depleted
Hexacosane	Fall armyworm gut	0.69	Depleted
(6E,10E,14E,18E)-2,6,10,15,19,23-hexamethyltetracosa-2,6,10,14,18,22-hexaene	Fall armyworm gut	0.06	Depleted
Octacosane	Fall armyworm gut	0.53	Depleted
(2R)-2,7,8-trimethyl-2-[(4R,8R)-4,8,12-trimethyltridecyl]-3,4-dihydrochromen-6-ol	Fall armyworm gut	2.96	Enriched
(3S,8S,9S,10R,13R,14S,17R)-17-[(2R,5R)-5,6-dimethylheptan-2-yl]-10,13-dimethyl-2,3,4,7,8,9,11,12,14,15,16,17-dodecahydro-1H-cyclopenta[a]phenanthren-3-ol	Fall armyworm gut	1.86	Enriched
(3S,4aR,6aR,6bS,8aS,12aS,14aR,14bS)-4,4,6a,6b,8a,11,11,14b-octamethyl-1,2,3,4a,5,6,7,8,9,10,12,12a,14,14a-tetradecahydropicen-3-ol	Fall armyworm gut	5.69	Enriched

Only metabolites detected in both the host plant and corresponding insect gut extract are shown. Full compound lists, including plant-only and gut-only compounds, raw GC-MS peak areas, normalization steps, log_2_ fold-change values, and additional calculations, are provided in the Supplementary Files. Fold change was calculated as the normalized mean peak area in the insect gut divided by the normalized mean peak area in the corresponding host plant. Values > 1 indicate enrichment and values < 1 indicate depletion.

**Table 2:** Fold-change comparison of shared metabolites in wheatgrass and desert locust gut extracts.

Compound Name	Insect Gut Sample	Fold Change	Status in Insect Gut
Methyl hexadecanoate	Male desert locust gut	1.02	Enriched
(3S,8S,9S,10R,13R,14S,17R)-17-[(2R,5R)-5-ethyl-6-methylheptan-2-yl]-10,13-dimethyl-2,3,4,7,8,9,11,12,14,15,16,17-dodecahydro-1H-cyclopenta[a]phenanthren-3-ol	Male desert locust gut	0.02	Depleted
(3S,8S,9S,10R,13R,14S,17R)-17-[(2R,5R)-5,6-dimethylheptan-2-yl]-10,13-dimethyl-2,3,4,7,8,9,11,12,14,15,16,17-dodecahydro-1H-cyclopenta[a]phenanthren-3-ol	Male desert locust gut	0.09	Depleted
Hexacosane	Male desert locust gut	0.74	Depleted
Pentacosane	Male desert locust gut	3.34	Enriched
(2E,7R,11R)-3,7,11,15-tetramethylhexadec-2-en-1-ol	Male desert locust gut	0.25	Depleted
(6E,10E,14E,18E)-2,6,10,15,19,23-hexamethyltetracosa-2,6,10,14,18,22-hexaene	Male desert locust gut	0.35	Depleted
(3S,8S,9S,10R,13R,14S,17R)-17-[(2R,5S,E)-5-ethyl-6-methylhept-3-en-2-yl]-10,13-dimethyl-2,3,4,7,8,9,11,12,14,15,16,17-dodecahydro-1H-cyclopenta[a]phenanthren-3-ol	Male desert locust gut	0.03	Depleted
Tricosane	Male desert locust gut	0.93	Depleted
Hexadecanoic acid	Male desert locust gut	1.49	Enriched
Methyl hexadecanoate	Female desert locust gut	0.87	Depleted
(3S,8S,9S,10R,13R,14S,17R)-17-[(2R,5R)-5-ethyl-6-methylheptan-2-yl]-10,13-dimethyl-2,3,4,7,8,9,11,12,14,15,16,17-dodecahydro-1H-cyclopenta[a]phenanthren-3-ol	Female desert locust gut	0.01	Depleted
Icosan-1-ol	Female desert locust gut	0.06	Depleted
(3S,8S,9S,10R,13R,14S,17R)-17-[(2R,5R)-5,6-dimethylheptan-2-yl]-10,13-dimethyl-2,3,4,7,8,9,11,12,14,15,16,17-dodecahydro-1H-cyclopenta[a]phenanthren-3-ol	Female desert locust gut	0.07	Depleted
Hexacosane	Female desert locust gut	2.07	Enriched
Pentacosane	Female desert locust gut	1.32	Enriched
(6E,10E,14E,18E)-2,6,10,15,19,23-hexamethyltetracosa-2,6,10,14,18,22-hexaene	Female desert locust gut	0.86	Depleted
(3S,8S,9S,10R,13R,14S,17R)-17-[(2R,5S,E)-5-ethyl-6-methylhept-3-en-2-yl]-10,13-dimethyl-2,3,4,7,8,9,11,12,14,15,16,17-dodecahydro-1H-cyclopenta[a]phenanthren-3-ol	Female desert locust gut	0.01	Depleted
Tricosane	Female desert locust gut	0.84	Depleted
Hexadecanoic acid	Female desert locust gut	0.32	Depleted

**Table 3:** Fold-change comparison of shared metabolites in mulberry and silkworm gut extracts.

Compound Name	Insect Gut Sample	Fold Change	Status in Insect Gut
(3S,4aR,6aR,6bS,8aS,12aS,14aR,14bS)-4,4,6a,6b,8a,11,11,14b-octamethyl-1,2,3,4a,5,6,7,8,9,10,12,12a,14,14a-tetradecahydropicen-3-ol	Male silkworm gut	0.20	Depleted
(2R)-2,7,8-trimethyl-2-[(4R,8R)-4,8,12-trimethyltridecyl]-3,4-dihydrochromen-6-ol	Male silkworm gut	0.30	Depleted
(9Z,12Z,15Z)-octadeca-9,12,15-trienoic acid	Male silkworm gut	7.70	Enriched
Methyl (9Z,12Z,15Z)-octadeca-9,12,15-trienoate	Male silkworm gut	0.01	Depleted
(3S,8S,9S,10R,13R,14S,17R)-17-[(2R,5R)-5,6-dimethylheptan-2-yl]-10,13-dimethyl-2,3,4,7,8,9,11,12,14,15,16,17-dodecahydro-1H-cyclopenta[a]phenanthren-3-ol	Male silkworm gut	0.34	Depleted
[(3S,10R,13R,14R,17R)-4,4,10,13,14-pentamethyl-17-[(2R)-6-methylhept-5-en-2-yl]-2,3,5,6,7,11,12,15,16,17-decahydro-1H-cyclopenta[a]phenanthren-3-yl] acetate	Male silkworm gut	0.13	Depleted
Methyl (9Z,12Z)-octadeca-9,12-dienoate	Male silkworm gut	0.03	Depleted
(2E,7R,11R)-3,7,11,15-tetramethylhexadec-2-en-1-ol	Male silkworm gut	0.02	Depleted
(6E,10E,14E,18E)-2,6,10,15,19,23-hexamethyltetracosa-2,6,10,14,18,22-hexaene	Male silkworm gut	0.07	Depleted
Hexadecanoic acid	Male silkworm gut	0.29	Depleted
(3S,4aR,6aR,6bS,8aS,12aS,14aR,14bS)-4,4,6a,6b,8a,11,11,14b-octamethyl-1,2,3,4a,5,6,7,8,9,10,12,12a,14,14a-tetradecahydropicen-3-ol	Female silkworm gut	0.57	Depleted
(2R)-2,7,8-trimethyl-2-[(4R,8R)-4,8,12-trimethyltridecyl]-3,4-dihydrochromen-6-ol	Female silkworm gut	0.06	Depleted
4,8,12,16-tetramethyloxacyclooctadecan-2-one	Female silkworm gut	1.16	Enriched
(9Z,12Z,15Z)-octadeca-9,12,15-trienoic acid	Female silkworm gut	12.26	Enriched
Methyl octadeca-9,12,15-trienoate	Female silkworm gut	0.05	Depleted
(3S,8S,9S,10R,13R,14S,17R)-17-[(2R,5R)-5,6-dimethylheptan-2-yl]-10,13-dimethyl-2,3,4,7,8,9,11,12,14,15,16,17-dodecahydro-1H-cyclopenta[a]phenanthren-3-ol	Female silkworm gut	0.68	Depleted
[(3S,10R,13R,14R,17R)-4,4,10,13,14-pentamethyl-17-[(2R)-6-methylhept-5-en-2-yl]-2,3,5,6,7,11,12,15,16,17-decahydro-1H-cyclopenta[a]phenanthren-3-yl] acetate	Female silkworm gut	0.47	Depleted
Methyl (9Z,12Z)-octadeca-9,12-dienoate	Female silkworm gut	0.06	Depleted
Methyl octadecanoate	Female silkworm gut	0.04	Depleted
(2E,7R,11R)-3,7,11,15-tetramethylhexadec-2-en-1-ol	Female silkworm gut	0.03	Depleted
(6E,10E,14E,18E)-2,6,10,15,19,23-hexamethyltetracosa-2,6,10,14,18,22-hexaene	Female silkworm gut	0.20	Depleted
Hexadecanoic acid	Female silkworm gut	0.31	Depleted

## Data Availability

The original contributions presented in this study are included in the Supplementary Material. Further inquiries can be directed to the corresponding authors. All data will be readily made available upon request, without hesitation.

## Supplementary Materials

The following supporting information can be downloaded at: https://www.mdpi.com/article/10.3390/metabo16060351/s1, GC-MS data, and python codes used to generate figures and tables seen in the main text.

## Abbreviations

The following abbreviations are used in this manuscript:

GC-MS	Gas Chromatography–Mass Spectrometry
NMDS	Non-Metric Multidimensional Scaling
UV–Vis	Ultraviolet–Visible Spectroscopy
